# Classification of protein motifs based on subcellular localization uncovers evolutionary relationships at both sequence and functional levels

**DOI:** 10.1186/1471-2105-14-229

**Published:** 2013-07-18

**Authors:** Marcos Parras-Moltó, Francisco J Campos-Laborie, Juan García-Diéguez, M Rosario Rodríguez-Griñolo, Antonio J Pérez-Pulido

**Affiliations:** 1 Centro Andaluz de Biologia del Desarrollo (CABD, UPO-CSIC-JA). Facultad de Ciencias Experimentales (Área de Genética), Universidad Pablo de Olavide, 41013, Sevilla, Spain; 2 Departamento de Economía, Métodos Cuantitativos e Historia Económica, Universidad Pablo de Olavide, 41013, Sevilla, Spain

## Abstract

**Background:**

Most proteins have evolved in specific cellular compartments that limit their functions and potential interactions. On the other hand, motifs define amino acid arrangements conserved between protein family members and represent powerful tools for assigning function to protein sequences. The ideal motif would identify all members of a protein family but in practice many motifs identify both family members and unrelated proteins, referred to as True Positive (TP) and False Positive (FP) sequences, respectively.

**Results:**

To address the relationship between protein motifs, protein function and cellular localization, we systematically assigned subcellular localization data to motif sequences from the comprehensive PROSITE sequence motif database. Using this data we analyzed relationships between localization and function. We find that TPs and FPs have a strong tendency to localize in different compartments. When multiple localizations are considered, TPs are usually distributed between related cellular compartments. We also identified cases where FPs are concentrated in particular subcellular regions, indicating possible functional or evolutionary relationships with TP sequences of the same motif.

**Conclusions:**

Our findings suggest that the systematic examination of subcellular localization has the potential to uncover evolutionary and functional relationships between motif-containing sequences. We believe that this type of analysis complements existing motif annotations and could aid in their interpretation. Our results shed light on the evolution of cellular organelles and potentially establish the basis for new subcellular localization and function prediction algorithms.

## Background

Proteins are responsible for performing the vast majority of cellular functions. Cells are highly compartmentalized, limiting most proteins to specific subcellular regions or organelles. The distribution of a protein plays a critical role in determining its activity [1]. For example, a DNA-binding protein would not be able to bind DNA if it were restricted to the endoplasmic reticulum (ER). Thus, knowing the subcellular localization of a protein gives us valuable information about its function.

The subcellular localization of large numbers of proteins has been determined experimentally. However, for many other proteins, both function and localization are unknown. Genomic approaches offer ways of rapidly predicting the subcellular localization of large sets of proteins [2]. Experimental and predicted localization data are compiled into curated protein databases such as Swiss-Prot [3], providing easy access to this biological information.

Another approach for predicting protein function is to look for the presence of sequences conserved with other proteins. Conserved sequences can be used to group proteins into families, which typically perform similar functions and share a common evolutionary ancestor. Motifs describe short amino acid arrangements that are shared by protein family members. They are designed to be used in conjunction with protein sequence databases to assign putative functions to unknown proteins. For example, the PROSITE database includes a comprehensive collection of mostly manually annotated motifs and is closely linked to the Swiss-Plot protein database [4]. Motifs in this database can be described as either patterns or profiles. Pattern motifs, or qualitative motif descriptions, use a consensus format while profiles, or quantitative motif descriptions, use scores for each amino acid position.

An ideal motif would identify all members of a protein family. However, due to the high degree of divergence between some protein family members, it is often difficult to define a motif that identifies all known family members without also identifying unrelated proteins. The threshold for positive sequence matches is usually determined by the chance of a particular motif occurring randomly. Depending on the stringency of the cutoff value used, a search may miss known family members or identify excessive numbers of unrelated proteins. To help evaluate motif specificity, PROSITE annotators use experimental evidence to assign positive hits as being true positives (TPs) if they are known to belong the protein class in question, or false positives (FPs) if not.

It has been suggested that the random emergence of conserved sequences may be subject to negative evolutionary pressure due to possible interference with the cellular function [5]. Different subcellular environments may have a role in allowing such sequences to emerge. Alternatively, motif conservation in FP proteins may not be random but instead reflect an evolutionary relationship with the protein family being studied. For example, a common ancestor may have diverged to perform different functions but retain common functional residues. Again, different subcellular environments may be an important factor in the evolution of divergent functions.

In this work, we have performed a computational analysis of motif sequence subcellular localization. As expected, we find that functionally-related TP sequences tend to be associated with specific subcellular localizations that are different to functionally-unrelated FP sequences. When multiple localizations are considered, TPs tend to be distributed between related cellular compartments, while FPs typically belong to unrelated compartments. Our analysis also identified cases where FPs are concentrated in particular subcellular regions, which may uncover relationships between functionally-unrelated sequences and give insight into the evolution of proteins in different cellular compartments.

## Results

### Identification of motif-containing protein sequences with known subcellular localizations

**Figure 1:**
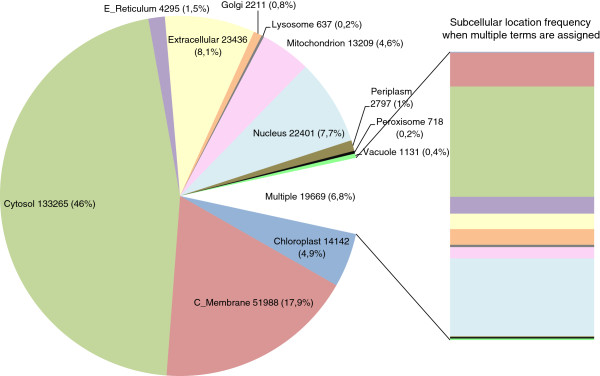
**Subcellular localization frequency of Swiss-Prot database proteins.** The number of sequences assigned to each localization is shown together with its relative percentage in brackets. The “Multiple” category represents the number of sequences with several localizations. The relative frequency of “Multiple” sequences for each localization is shown on the right bar using the same colors as the pie chart (see Additional file 3 and Additional file 4 for the complete list of proteins and assigned localizations).

We started with a dataset of 2344 profile and pattern sequence motifs. Each motif is linked to a set of true positive (TP) sequences (motif-containing proteins known to belong to the motif family), and a set of false positive (FP) sequences (proteins possessing the motif sequence but not its function). In order to analyze the subcellular localization data linked to each motif, we first assigned one or more subcellular localizations to each protein in the database (Figure 1) as detailed in the Methods section. The most frequently assigned compartments were the cytosol, followed by the cell membrane, secreted proteins (extracellular), and the nucleus, probably because they are the largest cellular compartments. When motifs with multiple subcellular localizations were considered, the frequency of the ER and Golgi apparatus (GA) increased significantly, consistent with their role as transition compartments for a large number of cytoplasmic, membrane-bound and secreted proteins.

**Figure 2:**
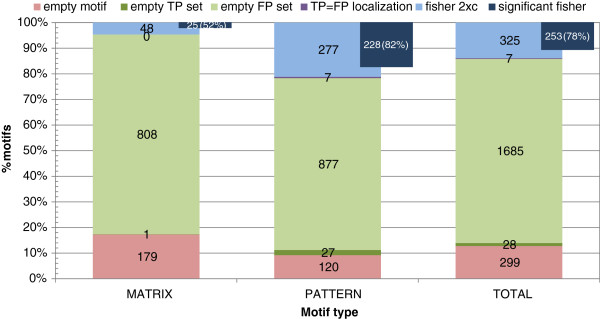
**Relative percentage of motifs with subcellular localization of sequences in TP and FP sets.** The number of motifs in each category, according to the results, is shown. The motifs are separated by matrices and patterns, and the total number is also shown in the latter bar. The categories are the following: *empty motifs*: neither TP nor FP sequences have been assigned any localization; *empty TP set*: no TP sequences have been assigned any localization; *empty FP set*: no FP sequences have been assigned any localization; *TP* = *FP localization*: both TP and FP sequences have only one localization which is the same; *fisher 2xc*: both TP and FP sequences have localization data. These latter motifs are suitable for analysis using Fisher’s exact test for 2xc tables to determine heterogeneity between TP and FP sequence localizations. The percentage of motifs with significant differences is shown overlapped with the Fisher 2xc bars, together with the corresponding relative percentage.

Next, we assigned subcellular localizations to both TP and FP protein sets for each motif. Using this method, one or more localizations were assigned to 60-61% of sequences (Table 1), although the TP and FP coverage for each motif was heterogeneous. Of the 2344 motifs analyzed, 299 had no subcellular localization data assigned to their sequences, with higher coverage in pattern versus matrix motifs (Figure 2). We found a low number of motifs (28) whose TP sequences had no subcellular localization data, of which virtually all were pattern motifs. In contrast, a high number of motifs (1685) completely lacked subcellular localization data for FP sequences. Finally, only 7 motifs had the same single subcellular localization for both TP and FP sequences, all of which were pattern motifs.

**Table 1:** Number of motifs in PROSITE and number of true and false positive proteins for each type of motif (Matrix/Pattern)

	**Motifs**	**TP**	**FP**
**Matrix**	1036	169463 (67%)	1151 (59%)
**Pattern**	1308	251645 (56%)	11788 (62%)
**Total**	2344	421108 (60%)	12939 (61%)

Percentage of sequences assigned subcellular localization is shown in brackets.

### Assignment of subcellular localizations to sequence motifs

We wanted to independently assign subcellular localizations to TP and FP sequence sets for each motif. To do this we compared the relative frequency of subcellular localizations in each set of TP and FP sequences against the frequency in the whole database. Only when a subcellular localization had a higher frequency in the set than the expected one (the frequency in the database) was it assigned to the motif. In this way we assigned one or multiple compartments (from 1 to 6) to 96% of the motifs with 1enough TP sequences for analysis. 69% of motifs were assigned a single subcellular localization, while 18% were assigned two different localizations (Table 2). The results for patterns and matrices were very similar.

**Table 2:** Frequency and percentage of subcellular localizations assigned to motifs

**Number of assigned SL**	**Matrix**		**Pattern**		**Total**	
	**Number of motifs**	**Percent**	**Number of motifs**	**Percent**	**Number of motifs**	**Percent**
**0**	46	5.37%	34	2.93%	80	3.97%
**1**	574	67.06%	824	70.97%	1398	69.31%
**2**	154	17.99%	221	19.04%	375	18.59%
**3**	59	6.89%	59	5.08%	118	5.85%
**4**	15	1.75%	18	1.55%	33	1.64%
**5**	7	0.82%	4	0.34%	11	0.55%
**6**	1	0.12%	1	0.09%	2	0.10%

The table only considers motifs with enough TP sequences for analysis (empty FP set, TP=FP localization, and fisher 2xc in Figure 2).

Next we tested if the subcellular localization of TP and FP sequences were significantly different (heterogeneous) from each other. To this end, the probability value for each motif was calculated using Fisher’s exact test for 2xc contingency tables. On average, 78% of the available motifs had a significant p-value (253 out of 325), indicating a high degree of heterogeneity between TP and FP compartments (Figure 2). Moreover, this heterogeneity was strongly related to pattern motifs, with 82% having a significant p-value versus 52% for matrix motifs (Figure 2).

**Figure 3:**
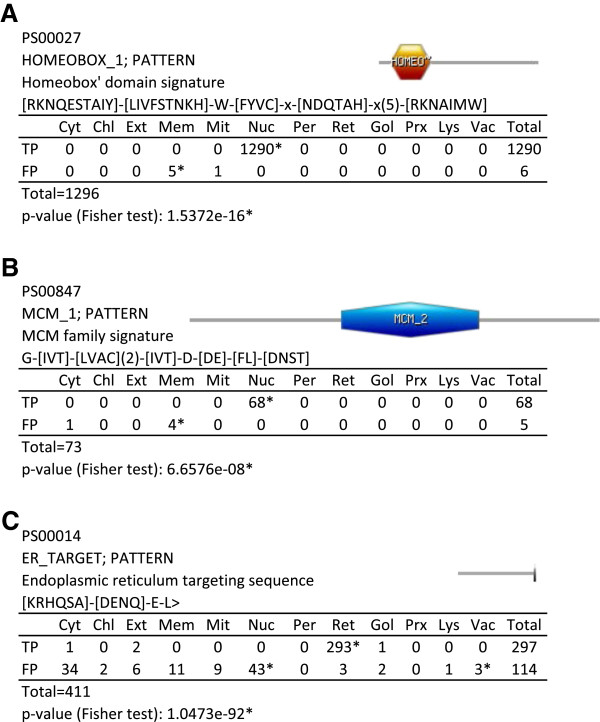
**Example tables with results for each motif.** For each motif: Accession, Description, Type (matrix or pattern), and consensus sequence is shown. Tables show the number of proteins annotated for each localization, and separated by TP and FP sequences. When a localization is considered significant for TP or FP it is marked with an asterisk. An asterisk is also used below when TP and FP results are found different by Fisher’s Exact test. These results were obtained using proteins with single subcellular localization (see Additional file 1 for results obtained using proteins with multiple localizations).

Once the calculations had been performed, a table summarizing our analysis was produced for each motif (example motif tables are shown in Figure 3). Each table independently lists the number of sequences assigned to each subcellular class for TP and FP sets, and highlights the most significant compartments. Tables for all the motifs can be found in Additional file 1 and Additional file 2. The p-value obtained from Fisher’s exact test is also shown.

### Distribution of motif sequences between related subcellular compartments

**Figure 4:**
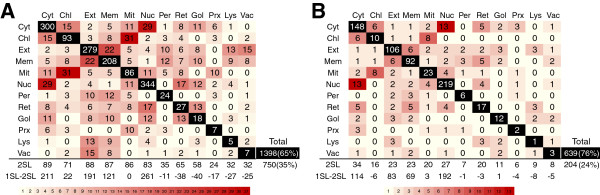
**Heat map with the number of motifs assigned to pairs of localizations.** Numbers represent the number of motifs assigned to two different localizations from higher (red) to lower (yellow) frequency. Numbers in black cells represent the number of motifs assigned to a single localization. **(A)** Results obtained using proteins with multiple subcellular localizations, and **(B)** results obtained using proteins with only single subcellular localizations. *Total*: total number (and percentage) of motifs assigned to single compartments, and total number (and percentage) of motifs assigned to multiple compartments; *2SL*: number of motifs for each localization when assigned together with each from the others; *1SL*-*2SL*: number of motifs assigned to a single localization minus those assigned to two separate localizations for each different category (black cell minus 2SL).

Given the high degree of interdependency between cellular structures and processes, we expected to find functionally-linked TP proteins in related compartments. About 19% of motifs have TP sequences distributed between two different subcellular classes (see Table 2). We tested these compartment pairs, and found that they were frequently linked (Figure 4A). The most frequent pairs were evolutionarily-related compartments such as mitochondrion and chloroplast, or compartments that share protein and molecular transit such as cytosol and nucleus or cell membrane and extracellular.

In some cases, multiple compartments were assigned to individual proteins. Thus, it is possible that our assignment of multiple subcellular localizations for individual motifs may be influenced by motif-containing proteins localized to multiple compartments. To test this possibility, we repeated our assignment of protein sequences to motifs but excluded sequences present in more than one compartment. The compartment pairs obtained in this way gave similar results to the previous analysis (Figure 4B), albeit with a lower number of pairs due to the reduced number of protein sequences used. In the second analysis, the ER appeared together with membrane and the cytosol, in addition to the nucleus. In fact, the ER, together with the GA, appeared linked to other compartments at a higher frequency than alone (1SL-2SL in Figure 4).

**Figure 5:**
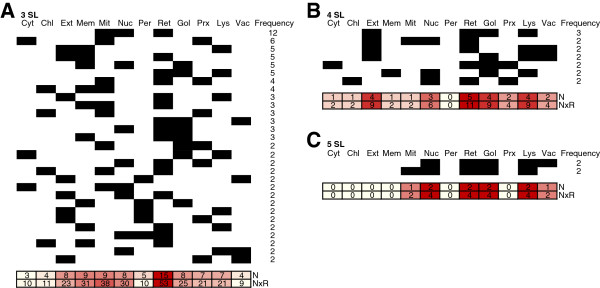
**Heat map with the number of motifs assigned to more than two localizations.** Each row shows a set of localizations (cells marked in black) when these are jointly assigned to the motifs: **(A)** 3 localizations, **(B)** 4 localizations, **(C)** 5 localizations. Numbers on the right side represent the number of different motifs assigned to this row, and numbers at the bottom represent the frequency that different combinations appear for each localization (N), and N multiplied by the number of repeats of each combination (NxR).

Next, we extended this analysis by looking at the relationship between the subcellular localizations of motifs assigned to more than two compartments. Compartment heat maps were generated for motifs with 3, 4, or 5 different TP localizations. The ER clustered with most other regions (Figure 5), consistent with its complex relationships with multiple cellular compartments.

### Non-random distribution of FP protein localization may indicate sequence convergence

**Figure 6:**
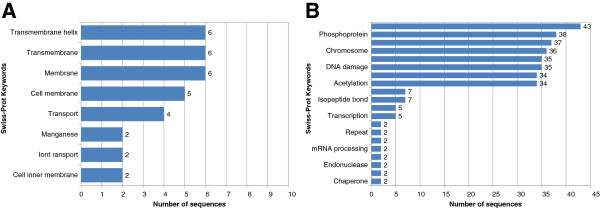
**Keywords found in FP sequences.** Swiss-Prot keywords with frequencies higher than one from groups of FP sequences: **(A)** Homeobox domain signature (6 FP sequences assigned to cell membrane and mitochondrion), where Transmembrane helix, Transmembrane and Membrane appear in all the sequences of this group; and **(B)** Endoplasmic reticulum targeting sequence (43 FP sequences assigned to the nucleus), where the Nucleus annotation appears in all the sequences of this group.

We have shown that TP and FP proteins have a strong tendency to differ in subcellular localization. This is expected given that true protein family members will generally be located in similar cellular regions to carry out their common functions. Conversely, if FPs are completely unrelated to the motif family and result from random sequence similarities, then we would not expect a strong bias in their subcellular distribution. However, we found several examples of motifs where FP sequences were concentrated in particular compartments. For example, the “Homeobox domain signature” motif (PROSITE:PS00027) was found in 1290 nuclear proteins (Figure 3A) where this pattern allows DNA binding through a helix-turn-helix type structure (PROSITE:PDOC00027). However, this motif was also found within 6 transmembrane proteins (false positives: 5 in the cell membrane and 1 in the mitochondrion membrane) with different known functions (Figure 6A). The homeobox motif overlaps a transmembrane region of 20 amino acids, according to the annotations in the Swiss-Prot database. It suggests that this motif has a different function in membrane-associated proteins. Another example, is the “MCM family signature” (PROSITE:PS00847) for minichromosome maintenance proteins involved in the initiation of ATP-dependent DNA replication. This pattern is a particular version of the B motif found in ATP-binding proteins, and is also found in 4 false positives from bacteria located in the cell inner membrane: 2 Xanthine phosphoribosyltransferases and 2 Glycerol-3-phosphate import ATP-binding proteins (Figure 3B). Again, it is likely that the motif of these latter 4 proteins arose independently during evolution due to the unrelated localization with respect to the nuclear true positives.

Motif sequences can occasionally be present in different cell compartments from where their associated function would indicate. In some cases this might suggest a common evolutionary origin. The “Endoplasmic reticulum targeting sequence” motif (PROSITE:PS00014) is a short C-terminal sequence (frequently with the four amino acids sequence: KDEL in vertebrates, or the consensus [HAD]DEL in yeasts) often found within proteins that accumulate in the lumen of the ER. We found this motif strongly linked to the ER, as expected, although some TP sequences also localize to other compartments. However, we also found FP sequences linked to the vacuole, where three proteins have this motif at their C-terminus (Figure 3C). We could hypothesize that the motif might still be involved in vesicle transport even though the proteins have not been reported as accumulating in the ER or they may play a modified but related function in the vacuole. Therefore, the C-terminal motifs in the FP sequences are likely to share a common evolutionary origin with the motif in TP sequences.

Interestingly, another 43 FP proteins with the same ER targeting motif (consensus [SQHA][QDEN]EL) at their C-terminus are localized to the nucleus and mainly involved in nucleosome biology and DNA repair (Figure 6B). The role of KDEL-like motifs in vesicle transport and ER retrieval has only been reported for cytoplasmic proteins and there is no evidence to link the function of these proteins to the nucleus. Thus, in contrast to the vacuolar proteins, it is unlikely that the motifs present in the nuclear FP sequences are evolutionarily related to the TP sequences.

In conclusion, the methodology presented in this work provides a rapid way of identifying motif-containing sequences associated with different cellular compartments that gives valuable information regarding the probable function of a motif and its evolutionary origin.

## Discussion

### The annotation of subcellular localization to motifs is easier with patterns than profiles

In this work we have systematically assigned subcellular localization information to both TP and FP motif sequences. PROSITE motifs are either pattern motifs, which use regular expression-like syntax, or matrix motifs, which use scores for each position in the motif sequence. We find that pattern motifs are better annotated than motif patterns. Only 48 matrix motifs have TP and FP sequences with annotated localizations versus 277 pattern motifs (Figure 2). In addition, pattern motifs allowed for better discrimination between TP and FP localizations. This suggests that sequence patterns are more robust than complex positional weight matrices for this type of analysis.

### Functionally-related TP sequences are localized to different compartments from FP sequences

We compared the subcellular compartments assigned to TP and FP motif sequences finding their distribution different for 78% of total motifs with sufficient localization data (52% of matrices, and 82% of patterns) (Figure 2). These results strongly link subcellular localization and function. It suggests that TP sequence motifs typically evolve in the context of particular cellular compartments, and are closely tied to these locations. Protein motifs are chosen because of their strong conservation and are usually key residues involved in protein function e.g. the active site of an enzyme or a protein-protein binding site. In some cases the link with localization may be directly related to function, such as a DNA binding protein that is functionally-linked to the nucleus. In other cases, the link with subcellular localization may be related to the local context of the protein partners necessary for function rather than the function itself.

### Subcellular distribution of TP motif sequences reflect functional or evolutionary relationships between subcellular compartments

We found a strong tendency for subcellular compartments to be related when we analyzed TP motif sequences associated to multiple localizations. Linked subcellular regions include compartments with significant protein exchange such as the cytosol and the nucleus, or compartments related by their origin, such as mitochondria and chloroplasts (Figure 4). Our findings are similar to other works where human proteins (not motifs) were classified by their subcellular localization [6]. The same authors also compared binary relations between compartments identified with the PSLT2 subcellular prediction method using yeast sequences [7]. Their results mostly correspond with the binary relationships we identified analyzing motifs. The exception is the plasma membrane and extracellular compartments. In contrast to their study, we did find these compartments frequently associated, which is what might be expected of compartments in direct contact.

One reason for the linked compartmentalization of motifs could be due to multiple localizations of individual proteins. However, when we repeated the analysis only using proteins with a single subcellular localization, we observed similar relationships between related compartments (Figure 4B). In addition, both the nucleus and the cytosol appear individually more than double compartments, while ER and GA motifs share localization with other compartments (Figure 4). This latter observation is not surprising considering the complex relationships between the ER, the GA and other parts of the cell.

The percentage of multi-compartmental proteins has previously been predicted to be at least 16% in humans [6]. We only found 6.8% of proteins in Swiss-Prot annotated as multi-compartmental according to their keywords (Figure 1). This value could be an underestimate due to incomplete annotation. However, the percentage is greatly increased (24-35%) when we take into account compartments assigned to motifs (Figure 4), suggesting a high level of multi-compartmentalization of protein motifs.

Biologically, this could suggest a common origin for motifs that appear in multiple compartments. If a new compartment emerges from another, the related proteins (and their motifs) would also be inherited, as occurs with the ER and GA [8] and mitochondria and chloroplasts (Figure 4). However, some of our data suggests that a common origin may not always result in the presence of common motifs. Although an endosymbiotic origin was suggested for peroxisomes [9], recent work based on both experimental evidence [10] and *in silico* analysis [11] has suggested that they are derived from the ER. It is therefore surprising that we did not find evidence for a binary relationship between peroxisomes and the ER, even though they were associated with mitochondria, chloroplasts and the cytosol. However, when more than two compartments were analyzed, peroxisome motif localization was almost equally related with ER, mitochondrion and chloroplast (Figure 5). In fact, it has been suggested that peroxisome proteins were recruited from eukaryotic compartments such as mitochondria and chloroplasts [12], which could explain these relationships.

Remarkably, some subcellular regions were more likely to contain motifs linked to multiple compartments than to them alone. For example, we found 65 and 58 examples, for the ER and GA, respectively, of motifs also associated with other compartments, versus 27 and 18 cases of a single compartment (Figure 4). Some compartments, especially the ER, showed a high frequency of motifs associated with multiple additional compartments (Figure 5). This is logical given that the ER is a compartment through which a large number of proteins are transported to other destinations. Some organelles, such as the GA and lysosomes, are in permanent dynamic equilibrium with the ER, from which they originate. The ER also establishes multiple contacts with most other intracellular organelles by means of narrow cytoplasmic gaps called membrane contact sites, including mitochondria, chloroplasts, the GA, the cell membrane, the nucleus, and lysosomes [13]. For example, organelles derived from endosymbiotic prokaryotes are not connected to the secretory pathway by vesicular traffic, meaning that mitochondria and chloroplasts acquire a large proportion of their lipids from the ER by non-vesicular routes [14]. Thus, polar lipid assembly in plants requires tight co-ordination between the chloroplast and the ER and necessitates inter-organelle lipid trafficking [15].

### Identification of possible functional or evolutionary relationships from the subcellular distribution of FP sequences

False positives are motif-containing sequences that have been assigned a known function that is distinct from the motif protein family. If FP motif sequence similarity is due to random sequence variation, with no functional or evolutionary connection with TP sequences, then we would not expect FP sequences to be linked to particular subcellular localizations in the same way as TP sequences. In fact, we identified several cases where FP sequences were strongly linked to specific subcellular compartments. Non-random distribution might suggest that the motif has functional significance in FP proteins. This could indicate sequence convergence if they arose independently from TP sequences or functional divergence if they shared a common ancestor.

For example, when we examined DNA-binding Homeobox domain motif proteins with single localizations, all TP sequences were restricted to the nucleus, while most FP sequences were assigned to the cell membrane and the mitochondrion (Additional file 1). It is very unlikely that membrane proteins have a DNA-binding function but it is also unlikely that they all possess this motif by chance. It may indicate that during the evolution of membrane proteins, the same motif has evolved independently to perform a different function by sequence convergence. In this case, there might be some kind of molecular or structural similarity with the DNA binding motif. DNA-binding domains have previously been found almost exclusively in nuclear proteins [16], but it is not the first time that homeobox domains have been linked with functions unrelated to DNA binding. The ceramide synthase protein LASS2 contains a homeodomain that has been implicated in V-ATPase protein binding, a proton-translocating pump located in the cytosolic membranes of vacuoles, lysosomes and the ER membrane [17].

Our analysis also revealed other possible examples of sequence convergence. The short ER targeting sequence motif, originally identified in proteins retained by the ER [18], also appears in a large number of nuclear FP proteins. Interestingly, this four amino acid motif always appears at the C-terminal end of both TP and FP sequences. Most of the nuclear sequences identified are fungal H2A histones (Figure 6B) which are not thought to pass through the ER. This strongly suggests that the ER targeting motif in the nuclear sequences has arisen independently through sequence convergence.

We also identified a number of vacuolar FP sequences with the ER targeting motif in their C-terminal domain. It was originally thought that the “Endoplasmic reticulum targeting sequence” permanently retained sequences within the ER but it is now known that it is required for the retrieval of proteins back to the ER following vesicular transport to other organelles [19]. Thus, it is possible that the motif might still have the ability to target proteins to the ER, but that either divergence from the KDEL motif or competing action from other protein sequences may have reduced its activity and allowed it to accumulate in other cellular compartments such as vacuoles. It is even possible that the ER targeting motif does, in fact, have a functional role in these proteins but that this has not yet been identified experimentally. In fact, the C-terminal KDEL sequence is found in some proteins transported by vesicles from the ER to vacuoles via a Golgi-independent route [20]. Determining the actual origin of these FP sequence motifs would require further analysis and/or experimentation but highlights the value of our methodology in identifying FP sequences of interest for further study.

### Systematic analysis of subcellular localization may help interpret motif annotations

The assignment of true and false positives is based on the available evidence, both of the actual function of the motif and of the individual sequences. The PROSITE database is composed of high quality manually-annotated motifs. Inevitably, these annotations need to be revised and updated periodically in response to new experimental evidence. Localization is likely to be an important line of evidence used by annotators when defining protein function for many motifs, especially in the case of motifs whose function is strongly linked to a particular subcellular organelle. This could be seen as a weakness in our approach because our analysis of subcellular localization may be using the same localization data employed by annotators to assign function to sequences. It is true that care must be taken when interpreting results for motifs whose function is strongly linked to localization. However, the previous example of the ER targeting motif highlights the potential difficulties of using localization to assign function. For example, experimental evidence may be incomplete or misleading. We would argue that a systematic summary of the subcellular localization of FP and TP sequences would aid both annotators and end users in interpreting the value of both a motif and the evidence used to assign function to TP and FP sequences.

## Conclusions

We have shown that protein sequence motifs are linked to related subcellular localizations, due in part to the evolutionary history of cellular compartments which has spatially restricted both motifs and their functions. Our results shed light on the evolution of functionally important sequences and the emergence of organelles. Systematically combining function and subcellular localization annotations has the potential to enhance our interpretation of sequence motifs. This methodology also lays the foundations for improved subcellular localization and function prediction algorithms.

## Methods

### Programming language

We wrote a program in the Perl programming language to perform all the calculations. Results are generated automatically and can be easily repeated simply by connecting the program to the latest motif and protein databases.

### Assignment of subcellular localization to protein sequences

We used Swiss-Prot release 2012_07 (July 11, 2012, http://www.uniprot.org/pub/databases/uniprot/previous_releases/release-2012_07/) and assigned one or more subcellular localizations to each protein sequence (Additional file 3) based on the following keyword terms (when the keyword is found, the localization in brackets is assigned): Cytoplasm (Cytosol: cyt), Chloroplast (Chloroplast: chl), Secreted (Extracellular: ext), Cell membrane (C_Membrane: Mem), Mitochondrion (Mitochondrion: Mit), Nucleus (Nucleus: Nuc), Periplasm (Periplasm: Per), Endoplasmic reticulum (E_Reticulum: Ret), Golgi apparatus (Golgi: Gol), Peroxisome (Peroxisome: Prx), Lysosome (Lysosome: Lys), and Vacuole (Vacuole: Vac).

A different dataset was also generated which excludes proteins with multiple related subcellular localizations which could distort the results (Additional file 4). This second dataset only contains proteins with single subcellular localizations. Note that it is possible that the cytosol annotation in the first dataset could include cytoplasmic organelles. However, when proteins with multiple localizations are excluded, the expected localization would be unequivocally cytosolic.

### Assignment of subcellular localization to PROSITE motifs

PROSITE is a reference database of sequence motifs. Motifs in this database can be described as either patterns or profiles. Pattern motifs use qualitative descriptions based on regular expression-like syntax (for example the N-glycosylation site pattern: N{P}[ST]{P} where the first position would be N, followed by any amino acid except P, then either S or T and finally any amino acid except P). On the other hand, profiles use quantitative motif descriptions, which employ scores for each amino acid position.

We used PROSITE release 20.83 (July 11, 2012) with 2344 motifs (http://www.expasy.org/databases/prosite/old_releases/prosite20_83.tar.gz).

Each PROSITE motif usually presents a set of true positive sequences (SWISS-PROT proteins with both the motif sequence and the assigned function of the motif), and a set of false positive sequences (SWISS-PROT proteins with only the motif sequence). We assigned subcellular localizations to each protein in TP and FP sets, and separately determined one or several subcellular localizations to each set using term enrichment. This analysis was performed by calculating the hypergeometric distribution in the following way: We have N sequences in the database, K of them contain localization X. We have n sequences in the TP (or FP) set. The probability that k sequences in the set will have the annotation X is:

$$P\left(x=k\right)\;=\frac{\left(\begin{array}{l} K\\ k \end{array}\right)\left(\begin{array}{l} N-K\\ n\;-k \end{array}\right)}{\left(\begin{array}{l} N\\ n \end{array}\right)}$$

If we assume that we have found L sequences with the localization X, the p-value represents the probability that we would find L sequences or more under the null hypothesis:

$$p-\text{value}\;=\;\sum_{k=L}^{\min\;\left\{K,n\right\}}p\left(x=k\right)$$

Thus, a p-value can be calculated for each localization in the TP or FP sets (independently for each set), and represents the probability of the sequences in this set of belonging to this subcellular localization. If this value is lower or equal to 0.05, it is considered significant (asterisks in Figure 3). Motifs with only one sequence (TP or FP) or all the TP and FP sequences bound to the same single subcellular compartment are discarded in this study due to insufficient data.

### Fisher test-based comparison of TP and FP subcellular localization

We wanted to know if TP and FP localizations are different (non-homogeneous) for each motif. That is, to know if the assigned subcellular localization for each protein in the TP set is significantly different to the subcellular localization assigned to the FP set. To this end, we used Fisher’s Exact Test for 2xc contingency tables (see [21], and the references within). This assessment of homogeneity tests the null hypothesis that the localizations for each motif are the same in both TP and FP sets. If the computed p-value is lower or equal to 0.05 the test is significant and therefore, the null hypothesis is rejected, meaning that the localizations in both sets are considered to be different. We used the FET2xc programs located at http://www.ugr.es/~bioest/software.htm, and were included in our analysis software.

### Annotation analysis

To analyze the annotations from the example motifs, Swiss-Prot keywords were extracted and counted for each protein and assigned to the significant subcellular localizations of the FP set. The keywords in “Technical term” (3D-structure, Reference proteome, Complete proteome) and “Coding sequence diversity” (Alternative splicing) groups were removed from the analysis to avoid irrelevant conclusions.

### Availability and requirements

**Project name:** analyzePrositeSL

**Project home page:** Additional file 5

**Operating system(s):** Linux

**Programming language:** Perl

**Other requirements:** HyGe.pm library (http://www.cs.huji.ac.il/course/2008/76552/Ex2/HyGe.pm) and FET2xc executables (http://www.ugr.es/~bioest/software.htm#MHD_y_2xc)

**License:** None for usage

## Abbreviations

TP: True positive; FP: False positive; ER: Endoplasmic reticulum; GA: Golgi apparatus.

## Competing interests

The authors declare that they have no competing interests.

## Authors’ contributions

MPM: carried out the majority of the study.

FJCL: carried out the majority of the study.

JGD: carried out the majority of the study.

MRRG: carried out the statistics analysis.

AJPP: conceived the study, carried out the design and coordination, helped in the computer programming, and wrote the manuscript.

All authors have read and approved the final manuscript.

## Supplementary Material

Additional file 1 Tables with results for each motif when proteins with multiple compartments are used (Motifs_annotation_multiple.xls). Click here for file

Additional file 2 Tables with results for each motif when proteins with a single localization are used (Motifs_annotation_unique.xls). Click here for file

Additional file 3 Subcellular localizations assigned to protein sequences in the SWISS-PROT database (Protein_localization_multiple.xls), column 1: SWISS-PROT identifier; column 2: subcellular localization (they are separated by commas if multiple localizations). Click here for file

Additional file 4 Subcellular localizations assigned to protein sequences in the SWISS-PROT database without including multiple localizations (Protein_localization_unique.xls), column 1: SWISS-PROT identifier; column 2: subcellular localization. Click here for file

Additional file 5 **Software for the subcellular localization assignment and the PROSITE motif analysis (analyzePrositeSL.pl).** To run the program, enter the following at the command line:./analyzePrositeSL.pl [PATTERN|MATRIX|1] [0|1] output_file where argument 1 is the motif type (1 = both), argument 2 represents the subcellular localizations analyzed (0 = seven localizations; 1 = twelve localizations), and the argument 3 is the main name for the output files. The output files are: file.mapping (Additional file 1 and Additional file 2), file.tables (Additional file 3 and Additional file 4), file.statistics (summary of several statistics from the results). Click here for file
